# The prevalence of stunting, overweight and obesity, and metabolic disease risk in rural South African children

**DOI:** 10.1186/1471-2458-10-158

**Published:** 2010-03-25

**Authors:** Elizabeth W Kimani-Murage, Kathleen Kahn, John M Pettifor, Stephen M Tollman, David B Dunger, Xavier F Gómez-Olivé, Shane A Norris

**Affiliations:** 1 MRC/Wits Rural Public Health and Health Transitions Research Unit (Agincourt), School of Public Health, Faculty of Health Sciences, University of the Witwatersrand, Johannesburg, South Africa; 2 Umeå Centre for Global Health Research, Department of Public Health and Clinical Medicine, Umeå University, Umeå, Sweden; 3 MRC Mineral Metabolism Research Unit, Department of Paediatrics, Faculty of Health Sciences, University of the Witwatersrand, Johannesburg, South Africa; 4 Department of Paediatrics, University of Cambridge, Cambridge, UK

## Abstract

**Background:**

Low- to middle-income countries are undergoing a health transition with non-communicable diseases contributing substantially to disease burden, despite persistence of undernutrition and infectious diseases. This study aimed to investigate the prevalence and patterns of stunting and overweight/obesity, and hence risk for metabolic disease, in a group of children and adolescents in rural South Africa.

**Methods:**

A cross-sectional growth survey was conducted involving 3511 children and adolescents 1-20 years, selected through stratified random sampling from a previously enumerated population living in Agincourt sub-district, Mpumalanga Province, South Africa. Anthropometric measurements including height, weight and waist circumference were taken using standard procedures. Tanner pubertal assessment was conducted among adolescents 9-20 years. Growth z-scores were generated using 2006 WHO standards for children up to five years and 1977 NCHS/WHO reference for older children. Overweight and obesity for those <18 years were determined using International Obesity Task Force BMI cut-offs, while adult cut-offs of BMI ≥ 25 and ≥ 30 kg/m^2 ^for overweight and obesity respectively were used for those ≥ 18 years. Waist circumference cut-offs of ≥ 94 cm for males and ≥ 80 cm for females and waist-to-height ratio of 0.5 for both sexes were used to determine metabolic disease risk in adolescents.

**Results:**

About one in five children aged 1-4 years was stunted; one in three of those aged one year. Concurrently, the prevalence of combined overweight and obesity, almost non-existent in boys, was substantial among adolescent girls, increasing with age and reaching approximately 20-25% in late adolescence. Central obesity was prevalent among adolescent girls, increasing with sexual maturation and reaching a peak of 35% at Tanner Stage 5, indicating increased risk for metabolic disease.

**Conclusions:**

The study highlights that in transitional societies, early stunting and adolescent obesity may co-exist in the same socio-geographic population. It is likely that this profile relates to changes in nutrition and diet, but variation in factors such as infectious disease burden and physical activity patterns, as well as social influences, need to be investigated. As obesity and adult short stature are risk factors for metabolic syndrome and Type 2 diabetes, this combination of early stunting and adolescent obesity may be an explosive combination.

## Background

Understanding the prevalence and patterns of undernutrition, particularly stunting, the emergence of overweight/obesity in children and adolescents, and the concomitant risk for metabolic disease, is of criticial importance for public health policy. Undernutrition is a serious risk factor for ill health and contributes substantially to the burden of disease in low- to middle-income countries (LMICs) [1]. Increasing adverse ramifications of childhood undernutrition are recognised later in life, and include impaired cognitive development, poorer educational achievement and human capital formation [2], and greater risk for obesity [3].

A nutrition transition, often accompanied by changes in physical activity levels, is being experienced in LMICs. Nutrition transition refers to changes in diet composition from traditional diets that are primarily derived from plant-based food sources low in fat and high in fibre, to more "Western" diets that are high energy dense and low in fibre. This transition is driven by rapid economic transition, urbanisation, globalisation, technological and social changes [4,5]. Nutrition transition typically begins with urban populations and those in higher social economic strata [4], but is not limited to these populations. Increased intake of animal source foods and edible oils have been documented in less urbanised urban areas and more urbanised rural areas [6].

Nutrition transition is a major driving force behind the double burden of malnutrition, a phenomenon that has become important in LMICs where high levels of obesity have been documented despite persistence of undernutrition [4,5]. Obesity has led to the increased public health importance of diet-related non-communicable diseases, such as cardiovascular diseases and diabetes, in LMICs particularly in adults [1]. In line with the co-existence of undernutrition and obesity, a protracted-polarised model of epidemiologic transition has been documented in LMICs. In this non-classical model of epidemiologic transition, infectious diseases and undernutrition coexist with non-communicable diseases and persist over prolonged periods of time [7]. Therefore, both undernutrition-related diseases, infectious diseases and obesity-related diseases contribute substantially to the burden of disease in these societies [1].

The problem of obesity and related metabolic disease risk is not only experienced among adults. Paediatric obesity has been documented in LMICs and is the driving force behind paediatric metabolic syndrome risk that has become a growing public health concern in LMICs [8]. Childhoood/adolescent obesity is associated with health problems for the child/adolescent including heightened risk of psychosocial morbidity, cardiovascular complications, and type 1 and type 2 diabetes [9]. Of further concern is the fact that obese children and adolescents are likely to be obese adults at increased risk of cardiovascular diseases and other morbidity, premature death, and impaired social, educational and economic productivity [3,9].

The World Health Organization (WHO) recommends more research into the frequency of risk factors related to the metabolic syndrome and their levels in LMICs [1]. Given that the pace and nature of transitions vary across geo-cultural settings, local data and context is increasingly stressed. This is particularly so because such information is essential to local programming and policy. The aim of this study is to investigate the prevalence of stunting and overweight/obesity by age and sex and to estimate the risk for metabolic disease in a group of children and adolesents aged 1-20 years randomly selected from a health and socio-demographic surveillance site in Agincourt, rural South Africa. We postulate that in rural South Africa stunting remains a concern among children, and that the transition to an urban profile with regards to overweight and obesity is advanced.

## Methods

### Study Setting and Population

This study was conducted in rural northeast South Africa, in the Agincourt sub-district, Mpumalanga province, alongside the country's border with Mozambique. Agincourt is a semi-arid setting, situated in the former Gazankulu homeland. The study was nested within the Agincourt health and socio-demographic surveillance system (HDSS), of the University of the Witwatersrand. Established in 1992 and covering the entire Agincourt sub-district, the Agincourt HDSS follows some 70,000 people living in 11,700 households in 21 contiguous villages. The population comprises Tsonga-speaking people, some 30% of whom are of recent Mozambican origin having entered South Africa mainly as refugees in the early to mid-1980s following the civil war in Mozambique. The Mozambicans are also Tsonga-speaking, have widely intermarried with the host South African population, and exhibit similar cultures.

The area is characterised by high levels of poverty: Mpumalanga province has one of the highest poverty rates in South Africa, at 64% [10]. There are high levels of unemployment: strict unemployment (excluding underemployment) is estimated at 29% for men and 46% for women [11]. Labour migration, mainly circular rural-urban migration, is widespread involving up to 60% of working age men and growing numbers of women [11]. Additionally, government support grants including the child support grant and pension for older people are an important source of income for many families. Being in a former homeland, the land is subdivided into plots too small to support subsistence farming. Housing material varies from traditional mud houses to brick houses. Piped water is available at community level, but there are frequent water shortages in most villages. Sanitation is poor, particularly in the former refugee settlements, with pit latrines of varying types from traditional pit latrines to ventilated improved pit latrines being the main method of excreta disposal in the area. Although the situation has improved in the last few years, roads are largely untarred and there is limited public transport with the main means of transport being privately owned taxis [12,13]. The area has benefited from the recent national electrification program. Access to education has improved in recent years: literacy levels have improved post-apartheid in the younger generation aged up to 29 years, but high illiteracy levels remain among the older generation, reaching levels of almost 80% for those aged 60 years and above [11]. Health care services are limited in the area: a network of five primary care clinics refers to a larger public health centre; the nearest district hospital is 25 kilometres away. The area is characterised by a high prevalence of HIV/AIDS, slightly over 30% among pregnant women visiting public antenatal health clinics in the province [14]. The study area, Agincourt HDSS and local demographics are described in detail elsewhere [12].

### Data collection

The study was conducted between April and July 2007. The Agincourt HDSS, a longitudinal community surveillance system acted as the sampling frame for the study. It involves a systematic annual recording of vital demographic events including births, deaths and migrations occurring in all households in 21 contiguous villages in Agincourt sub-district. The baseline census was conducted in 1992 and data are updated annually. The study sample comprised children and adolescents aged 1-20 years selected from the entire population within this age spectrum in the Agincourt HDSS as at March 2007 (n = 34775; 50% boys and girls respectively). For analysis and documentation purposes, the ages were truncated to full years; thus, for example, 20 years refers to participants aged 20.0-20.9 years. Four thousand children and adolescents were targeted, comprising 100 males and 100 females for each year of age. We oversampled 10-15 children per age-sex group to counter possible non-participation. Thus a total of 4658 children were randomly selected from the Agincourt HDSS database. Only children who had lived in the study area at least 80% of the time since birth, or since 1992 when enrolment into the Agincourt HDSS began, were included. A random sample of children was drawn from each age-sex-village stratum in proportion to the population size of the village.

Written informed consent was obtained from the parent/caregiver of children aged 1-17 years and from adolescents 18-20 years themselves. Consent was obtained duringl household visits on weekdays, and participants were invited to data-collection camps over the weekends and holidays at centrally located schools within the study villages. Assent was also obtained from those aged 9-17 years prior to data collection. Ethical clearance was granted by the University of the Witwatersrand Committee for Research on Human Subjects, Medical (M070244).

Anthropometric measurements were carried out on all children and adolescents aged 1-20 years, and pubertal assessments were done on those 9-20 years old. Height was measured using a stadiometer (Holtain, UK) calibrated in millimeters. For all children aged less than 24 months, the determination of length was done using an inelastic tape measure in a recumbent position on a flat surface. Weight in kilograms (to one decimal point) was determined using a mechanical bathroom scale (Hanson, UK). Waist circumference was measured in milimetres using an inelastic tape measure at the natural waist (midway between the tenth rib and the illiac crest) with the participant in a standing position. All measurements were carried out according to standard procedures [15].

Pubertal assessment in children 9 years and older was obtained using the Tanner 5-point pubertal self-rating scale which has been validated for Black South Africans [16]. This self-administered questionnaire was conducted for males and females separately, with the assistance of same-sex interviewers who explained how to complete the assessment prior to the participants completing the questionnaire. The five Tanner stages reflect physical development based on external primary and secondary sex characteristics: pubic hair in girls and boys, breast development in girls and genitalia in boys [17]. Genital development in boys and breast development in girls were used to define the stages in this study.

### Data quality

A team of 12 fieldworkers was carefully trained by experts in anthropometric measurements. To minimise fieldworker variation, each fieldworker specialised in a specific measurement and collected the data on all study participants. Coefficient of variation for anthropometric measurements was determined before and towards end of the study period; on average, the coefficient of variation towards the end of the study ranged between <1% and 3% for the different measurements.

### Data analysis

Weight-for-age z-scores (WAZ), height-for-age z-scores (HAZ) and weight-for-height z-scores (WHZ) for children up to 60 completed months were generated using the 2006 World Health Organization (WHO) growth standards with the WHO Anthro 2005 program, Beta Version [18]. Z-scores for those aged 5-17 years were determined using the 1977 National Center for Health Statistics (NCHS)/WHO reference. For the WHO standards, weight-for-length standards range from 45 to 110 cm (for children aged younger than 24 months) while weight-for-height standards (for children 24-60 months) range from 65 to 120 cm for both sexes. For the NCHS/WHO reference, weight-for-length reference ranges from 49 to 103 cm and weight-for-height 55 to 145 cm for boys (up to 11 years approximately); while for girls, weight-for-length reference ranges from 49 to 101 cm and weight-for-height 55 to 137 cm (up to 9 years approximately). Thus, for the purpose of comparisons by sex, WHZ scores were calculated for all children up to 9 years.

Overweight and obesity in children 2-17 years were determined using the absolute age and sex specific cut-offs for body mass index (BMI) recommended by the International Obesity Taskforce (IOTF) [19]. These are defined to pass through BMI of 25 and 30 kg/m^2 ^at 18 years for overweight and obesity respectively. For adolescents 18-20 years, adult cut-off points of BMI ≥ 25 and ≥ 30 kg/m^2 ^for overweight and obesity respectively, were used [20].

Studies have shown that waist circumference is a better predictor of child and adolescent risk of metabolic disease than BMI, or improves the ability of BMI in predicting this [21]. We used waist circumference alone and in combination with height in estimating the metabolic disease risk [22]. Waist-to-height ratio (WHtR) was generated by dividing waist circumference by height. The percentage of those at risk of metabolic disease was estimated among adolescents who had attained at least Tanner stage 3 as it was assumed that these had attained approximately adult height. This was done using cut-offs of waist circumference of ≥ 94 cm for males and ≥ 80 cm for females [23], and waist-to-height ratio of 0.5 for both sexes [22].

Data analysis was done using Stata version 10.0 (StataCorp LP, College Station, Texas, USA). Patterns of the prevalence of stunting (HAZ <-2), underweight (WAZ <-2), wasting (WHZ <-2), overweight and obesity by age and sex were determined. The Student t-test was used to test the differences between means across age-sex groups, and the chi-square test for differences in proportions by age and sex. A p-value of < 0.0500 was considered statistically significant.

## Results

Nearly 80% (3511 participants) of the randomly selected sample participated in the study. Non-participation was due to failure to present for measurements after giving consent (9%), refusal to consent (1%), absence due to being in boarding school (8%), out-migration from study area (3%), and being away for other reasons (3%). From the 3511 participants, 22 were excluded from the analysis: pregnant adolescents (n = 9), severely mentally and physically disabled children (n = 11), one case with spurious date of birth, and one case with erroneous measurements. A total of 3489 children were included in the analysis: 1724 (49.4%) males and 1765 (50.6) females aged between 1-20 years. Distribution of participants included in the analysis by sex and year of age is shown in Table 1. Further, the distribution of adolescents 9-20 years by Tanner pubertal stages is shown in Table 2. Most boys were in stages 1 and 2 while most girls were in stages 3-5. Only 5% of boys had reached stage 5.

**Table 1:** Distribution of study participants aged 1-20 years (n = 3489), by age and sex, Agincourt sub-district, South Africa, 2007

Age (mean)	Boys		Girls	
	**N**	**%**	**N**	**%**
1(1.6)	70	4.1	67	3.8
2(2.5)	83	4.8	77	4.4
3(3.5)	103	6.0	91	5.2
4(4.5)	82	4.8	98	5.6
5(5.5)	87	5.1	86	4.9
6(6.5)	104	6.0	94	5.3
7(7.5)	91	5.3	110	6.2
8(8.6)	111	6.4	97	5.5
9(9.5)	90	5.2	100	5.7
10(10.5)	98	5.7	101	5.7
11(11.5)	105	6.1	97	55
12(12.5)	96	5.6	89	5.0
13(13.5)	94	5.5	97	5.5
14(14.5)	78	4.5	89	5.0
15(15.5)	82	4.8	99	5.6
16(16.5)	74	4.3	94	5.3
17(17.5)	69	4.0	67	3.8
18(18.5)	80	4.6	63	3.6
19(19.5)	70	4.0	67	3.8
20(20.6)	57	3.3	82	4.7

	1724		1765	

**Table 2:** Distribution of adolescents aged 9-20 years by sex and pubertal stage (n = 2006), Agincourt sub-district, South Africa, 2007.

	Boys		Girls		Significant difference by sex (P-value)
	
Stage	Mean age (SD)	N (%)	Mean age	N (%)	
1	11.3 (1.5)	254 (26)	10.5 (1.1)	156 (15)	P < 0.0001
2	13.3 (2.4)	296 (30)	12.0 (1.7)	186 (18)	P < 0.0001
3	16.4 (2.3)	188 (19)	14.6 (2.4)	243 (24)	P < 0.0001
4	18.1 (1.9)	193 (20)	16.9 (2.3)	306 (30)	P < 0.0001
5	18.5 (1.8)	45 (5)	18.3 (2.0)	139 (14)	P = 0.3954

Total	14.1 (3.3)	976(49)	14.1 (3.4)	1030(51)	

### Prevalence of stunting, underweight and wasting by age and sex

**Figure 1:**
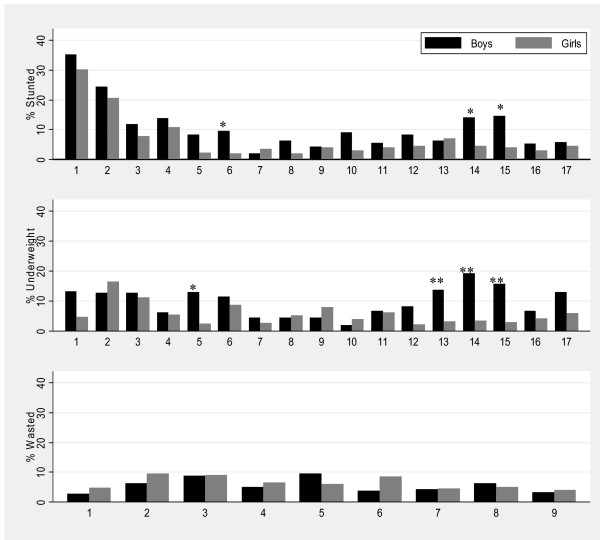
**Prevalence of stunting and underweight for children aged 1-17 years (n = 3070) and wasting for children aged 1-9 years (n = 1641) by sex, Agincourt sub-district, South Africa, 2007**. (Significant difference by sex: *P-value < 0.05, **P-value < 0.01, ***P-value < 0.001).

Figure 1 shows the prevalence of stunting, underweight and wasting for boys and girls at each age, with the level of significance by sex indicated by an asterisk on the bars. The prevalence of stunting fell from 32% at 1 year to plateau at approximately 3-6% from 5 years, before rising to 14-15% in boys during adolescence between years 14 and 15. Stunting was significantly greater in boys than girls at 6, 14 and 15 years (p < 0.0500 respectively). Stunting was the most prevalent form of undernutrition for younger children aged 1-4 years at 18% (Table 3).

**Table 3:** Comparison of Prevalence of malnutrition in current study with national studies in South Africa

Study	Population (n)	Reference		Stunted %			Underweight %			Wasted %			Overweight %			Obese %		
				**U**	**R**	**N**	**U**	**R**	**N**	**U**	**R**	**N**	**U**	**R**	**N**	**U**	**R**	**N**
			
SAVACG (1994)	6-71 mo (11430)	WHO/NCHS		16	27	23	7	11	9	2	3	3						
NFCS (1999)	1-9 y (2894)	WHO/NCHS IOTF		17	27	22	8	13	10	2	5	4	13	12	12	6	4	5
YRBS (2002)	Approx. 13-19 y (9224)	WHO/NCHS IOTF		-	-	11	-	-	9	-	-	4	-	-	17	-	-	4
DHS (2003)	<5 y (1159)	WHO/NCHS		27	28	27	12	11	12	6	5	5	-	-	-	-	-	-
NFCS (2005)	1-9 y (2469)	WHO/CDC IOTF		16	20	18	9	9	9	5	4	4	10	10	10	4	4	4
Agincourt (2007)	1-20 y (3489)	WHO WHO/NCHS IOTF		-	8	-	-	7	-	-	6	-	-	6	-	-	2	-
	1-4 y (671)			-	18	-	-	10	-	-	7	-		7	-	-	1	-
	5-9 y (970)			-	5	-	-	6	-	-	6	-		4	-	-	1	-
	10-14 y (944)			-	7	-	-	7	-	-	-	-		6	-	-	2	-
	15-20 y (904)			-	6	-	-	8	-	-	-	-		8	-	-	4	-

U = Urban; R = Rural; N = National

Sources:

SAVACG (1994): The South African Vitamin A Consultative Group, 1995 [29]

NFCS (1999): Labadarios et al., 2005 [28]

YRBS (2002): Reddy et al., 2008 [41]

DHS (2003): Department of Health, 2003 [27]

NFCS 2005: Department of Health, 2007 [26]

Agincourt (2007): Current study

The prevalence of underweight in younger children ranged between 6 and 14% for children 1-6 years, was lower for children between ages 7 and 12 years, and peaked at 19% in boys aged 14 years. The prevalence of underweight was significantly higher in boys than in girls at ages 5, 13, 14 and 15 (p < 0.0500).

The prevalence of wasting was uncommon at age 1 year but increased to approximately 4-9% between 2-9 years. There was no difference in wasting by sex.

### Prevalence of stunting, underweight and wasting by Tanner pubertal staging and sex

**Figure 2:**
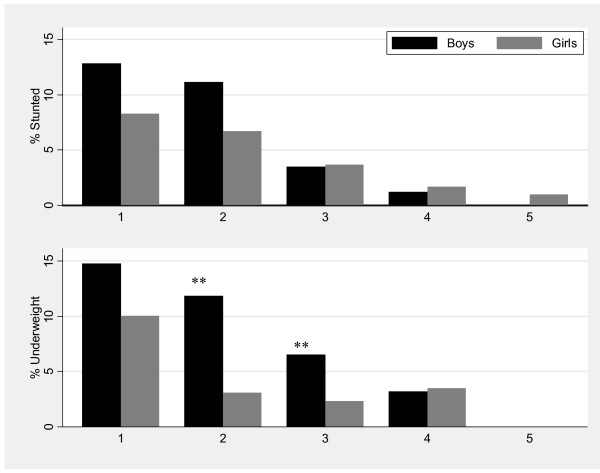
**Prevalence of stunting and underweight for adolescents aged 9-20 years (n = 2006) by Tanner stage and sex, Agincourt sub-district, South Africa, 2007**. (Significant difference by sex: **P-value < 0.01).

Figure 2 shows the prevalence of stunting, underweight and wasting in children at Tanner stages 1-5, with the level of significance by sex indicated by an asterisk on the bars. There was no significant difference in stunting by sex at the various Tanner stages. Stunting was highest at Tanner stage 1 for both girls and boys at an average of 9%, and reduced with increasing stage to about 1% at Tanner stage 5. Boys at Tanner stage 2 and 3 were significantly more underweight than girls at the same stage; 11% vs. 4% (p = 0.0080) and 10% vs. 2% (p = 0.0010) respectively.

### Prevalence of overweight and obesity by age and sex

**Figure 3:**
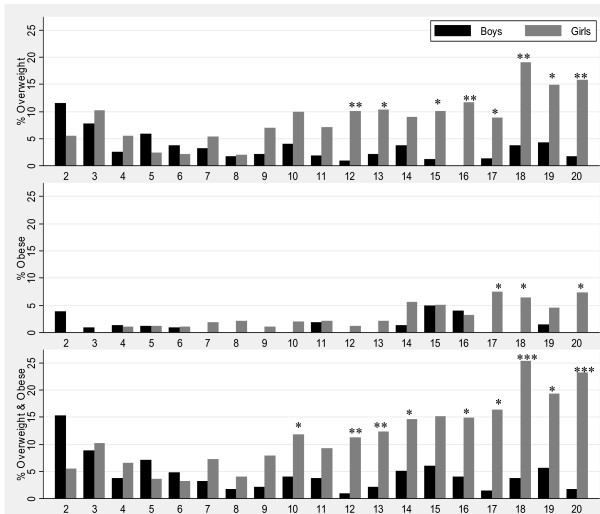
**Overweight and obesity for children aged 2-20 years (n = 3358) by sex, Agincourt sub-district, South Africa, 2007**. (Significant difference by sex: *P-value < 0.05, **P-value < 0.01, ***P-value < 0.001).

Figure 3 shows the prevalence of overweight and obesity by age and sex with the level of significance by sex indicated with an asterisk on the bars. The prevalence of overweight and obesity was moderate in early childhood and low in late childhood, and remained so in older boys. However, the prevalence rose progressively in girls aged 10 years and older. Consequently, girls had significantly higher prevalence of combined overweight and obesity (p < 0.0500) than boys at ages 10 and 12-20 years; at age 15 years, significance was borderline (p = 0.0500). From age 14 years, overweight and obesity averaged some 18% in females compared to 4% in males, reaching approximately 20-25% in late adolescence in girls.

Co-existence of stunting and combined overweight and obesity in the same child was common in children aged less than five years (18%), but was uncommon in older children aged 5-9 years (5%) and adolescents aged 10-20 years (3%).

### Prevalence of overweight and obesity by Tanner stages

**Figure 4:**
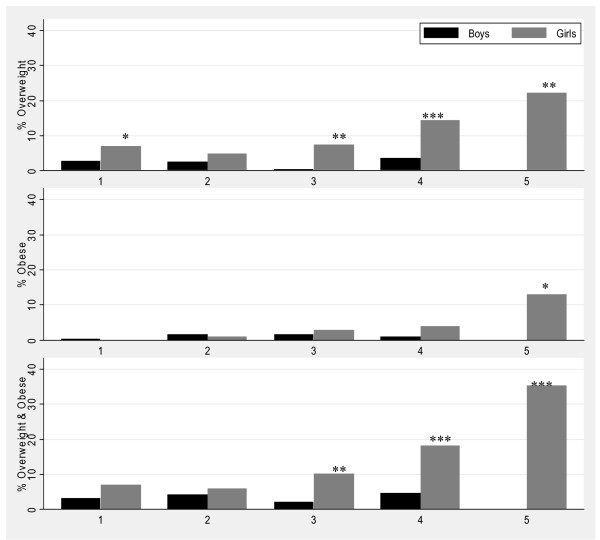
**Overweight and obesity for adolescents aged 9-20 years (n = 2006) by Tanner pubertal staging and sex, Agincourt sub-district, South Africa, 2007**. (Significant difference by sex: *P-value < 0.05, **P-value < 0.01, ***P-value < 0.001).

Figure 4 depicts the relationship between the prevalence of overweight and obesity with Tanner pubertal stage in girls and boys. Combined overweight and obesity in girls was low in the earlier stages of puberty, but increased markedly during the later stages (from 7% to 35%); while in boys the prevalence remained low throughout the stages. The prevalence of combined overweight and obesity was significantly different between males and females at Tanner stages 3, 4 and 5 (p < 0.0010 respectively).

### Risk for metabolic disease

**Figure 5:**
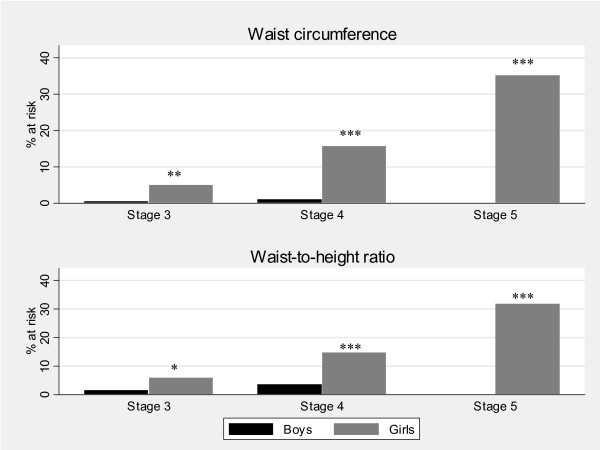
**Metabolic disease risk using waist circumference and waist-to height ratio cut-offs for adolescents in tanner stages 3-5 (n = 1114) by Tanner stage and sex, Agincourt sub-district, South Africa, 2007**. (Significant difference by sex: *P-value < 0.05, **P-value < 0.01, ***P-value < 0.001).

Risk for metabolic disease, defined by central obesity using waist circumference and WHtR cut-offs [22,23] among adolescents at Tanner stages 3-5, is presented by sex and pubertal stage in Figure 5. Using the waist circumference cut-offs, about 10% of adolescents were potentially at risk of metabolic disease; significantly higher proportion of girls (16%) than boys (1%) (p < 0.0001). Similarly, using the waist-to-height cut-offs, 10% of adolescents were potentially at risk with significantly higher proportion of girls (15%) than boys (3%) (p < 0.0001). The prevalence of risk using the different cut-offs increased with increasing pubertal stage in girls: 5% in girls at Tanner stage 3 using the waist circumference cut-offs, increasing to 35% at Tanner stage 5. There were significant differences by sex across all the Tanner stages using the waist circumference and WHtR cut-offs (p < 0.05 at all stages respectively). Surprisingly, no boys were at risk of metabolic disease in Tanner stage 5 using the two cut-offs. However, there were fewer boys (n = 45) who had attained this pubertal stage compared to girls (n = 139).

## Discussion and Conclusions

This study presents evidence of a double-pronged problem of malnutrition; undernutrition and overweight/obesity in children and adolescents living in a rural South African community. This phenomenon is evident in societies undergoing nutrition transition in LMICs [4,5]. This study also documents noteworthy levels of overweight and obesity and central obesity among adolescent girls, indicating an elevated risk for metabolic disease. These findings are relevant to the heightened public health interest in paediatric metabolic syndrome in LMICs [8]. Both undernutrition and overnutrition traverse the life course and are important both at the individual and national level [2,9,24]. Further, both paediatric obesity and adult short stature are risk factors for the metabolic syndrome and Type 2 diabetes in adulthood [9,25]. Hence the combination of early stunting and adolescent obesity raises critical concerns. The study calls for urgent evidence-based policy development and interventions to address the dual problem of malnutrition in rural South Africa.

The levels of undernutrition in young children in this rural population correspond with earlier findings in South Africa [26-29] (see Table 3). They indicate the persistence of undernutrition in rural communities despite recent efforts to address household food insecurity, in part through the introduction of child support grants and old age pensions. Despite South Africa being a middle-income country, food insecurity remains a problem with some 35% of households considered food insecure [30]. The Agincourt area is typical of former apartheid homeland areas with a limited food production base. Located in geographically inhospitable areas, land is sub-divided into plots that are generally too small to support subsistence agriculture. This results in people having to rely substantially on purchased food which - in an area where people have low purchasing power - may result in food insecurity and consequent undernutrition. The poor nutritional outcomes shown in our study participants may not only be associated with the quantity of food but also the quality. Food variety and dietary diversity, which are associated with nutritional status of South African children, are limited in poor communities in South Africa [31]. The potential role of HIV/AIDS in the persistence of undernutrition in our study community cannot be underestimated. The high prevalence of HIV among women of child bearing age [14] indicates that many households are HIV affected. HIV/AIDS has an extensive effect on food security as it undermines the ability of households to provide for their basic needs [32]. Research in the study area shows that adult mortality, particularly that of a male wage-earner, affects household food security and that people often fall back on wild foods as a coping strategy [33]. Death of a mother has been associated with a fourfold increase in the odds of child undernutrition in the study area [34].

The results show higher prevalence of undernutrition amongst adolescent boys compared to girls. This confirms findings on adolescents in other South African studies [35] and on younger children elsewhere in sub-Saharan Africa [36]. The differential prevalence of undernutrition by sex in our study is likely to be due to delay in the pubertal growth spurt in boys compared to the reference group. This occurs where undernutrition is prevalent [37]. Other studies in South Africa have documented delayed pubertal development for children in rural areas compared to their urban counterparts [38]. Other factors may contribute to these differences because even after stratifying by pubertal stage, boys at Tanner stages 2 and 3 still showed significantly higher levels of underweight compared to girls in the same stage. Evidence amongst younger children indicates that disproportionate male undernutrition occurs in households with low socio-economic status; in better off households, the sex difference disappears [39]. Further investigations into the sex differences of malnutrition during adolescence in our study may be required.

In addition to the substantial prevalence of undernutrition documented in this study are noteworthy levels of overweight and obesity among adolescents, particularly girls. The levels of overweight/obesity are comparable to those documented in other rural areas in South Africa [40]. These levels are, however, lower than those recorded in various national surveys (Table 3) [26,28,41], indicating that there may be areas with very high levels. The South African Youth Risk Behaviour Survey 2002 documented a prevalence of combined overweight and obesity of 21% among adolescents in grades 8-11 (approx 13-19 years) nationally; 7% in boys and 25% in girls [41]. Our study documented a prevalence of about 10% in this age group; 4% in boys and 16% in girls.

As with overweight and obesity, prevalence of central obesity was substantial particularly amongst adolescent girls. Central obesity, measured using waist circumference alone or in combination with other measures, is an integral risk indicator for metabolic syndrome [23]. The risk in this study increased with sexual maturation, indicating higher risk as the adolescents transition to adulthood. The finding of a substantial level of central obesity is important as childhoood/adolescent metabolic syndrome risk is increasingly becoming a concern in LMICs [8]. Childhoood/adolescent overweight/obesity is associated with many health problems for the child/adolescent including heightened risk of psychosocial morbidity, asthma, orthopaedic difficulties, cardiovascular complications, and type 1 and type 2 diabetes [9]. Further, overweight/obese children and adolescents are likely to be obese adults at increased risk of cardiovascular and other morbidity, premature death, and impaired social, educational and economic productivity [3,9]. Risk components for the metabolic syndrome have been tracked from childhood to adulthood in several studies [42]. This emphasises the importance of identifying these risk factors and addressing the problem early during childhood to prevent transfer of these risks to adulthood.

The higher prevalence of obesity among adolescent girls compared to boys in this study is in keeping with many studies in other LMICs [43] and in South Africa in particular [35]. Several factors may explain these sex differences. Biologically, energy needs differ for boys and girls and also in relation to rate of growth. Further, timing of sexual maturation differs by sex [44]. Behavioural factors are also important in explaining the sex differences: boys are generally more physically active compared to girls especially during adolescence [45,46]. Concerns about body image, particularly among adolescent girls, may lead to problematic eating behaviours such as irregular meal patterns which may result in increased weight gain [47]. Differential problematic eating behaviours by sex have been reported among South African youth [48].

The phenomenon of a double burden of malnutrition documented in this study is becoming increasingly important in LMICs undergoing nutrition transition [4,5]. Several factors shed light on why this phenomenon is occuring in rural South Africa. Literature on early developmental programming describes how nutritional deprivation during the foetal period and early childhood leads to adaptations that may result in obesity during later life [3]. This may partly explain the co-occurrence of stunting in early childhood with overweight and obesity during adolescence in our study. Further, change of food cultures and lifestyle may also play a major role. Studies on urbanisation in South Africa have reported decreased intake of staple foods including maize meal, and increased intake of energy-dense foods including added fats and oils and animal-derived foods [49]. Similar findings have been documented in other LMICs undergoing nutrition transition [4]. Over-reliance on energy-dense processed foods, purchased due to insufficient local food production, may be a key factor in the development of adolescent overweight and obesity in the study area. Extensive labour migration to larger towns [11] facilitates the transfer and introduction of urban practices to rural settings with consequent change in diet and lifestyle. Women's participation in the labour force, increasingly reported in the study area, may also impact on food supply and diet [11].

Physical inactivity and sedentary lifestyles are associated with childhood/adolescent overweight and obesity [50]. National electrification in South Africa in the last few years, with consequent increase in televisions at home, may have resulted in decreased physical activity. Studies in South Africa have reported decreased physical activity among adolescents [45,46]. Increased television viewing by children/adolescents may also be associated with increased consumption of unhealthy foods seen in television advertisements [51]. Length of television viewing by children/adolescents has been associated with higher consumption of fast foods and other high energy dense food and lower intake of fruits and vegetables [52]. Contribution of these factors to the patterns of obesity we have observed need further investigations.

Despite the important findings in our study, a few limitations should be noted. This study did not collect data on food intake and physical activity patterns which would help explain the findings. However, further work is underway to determine factors associated with nutritional status in the study area, including dietary patterns and physical activity levels. Owing to the Agincourt HDSS sampling frame used, there were no infants in the study sample. This may have implications for findings, particularly with regards to undernutrition. There was higher non-participation among older adolescents. This may indicate over-representation of the study sample by younger children and may have implications for the overall findings. Despite these limitations, it is important to point out the strength of the sampling procedure employed. The sampling was purely random, using an existing sampling frame, and representative of the study area as the study sample was drawn from the various villages in proportion to the population size of the villages. The findings provide useful picture of patterns of nutritional status among children and adolescents in rural South Africa. They also raise important questions that could be followed up in future studies.

Our findings have implications at individual, community and national levels that traverse the life course. The importance of both paediatric undernutrition and overweight/obesity cannot be overemphasised [1,2,8]. The overweight/obesity prevalence we have observed in this study, particularly in adolescent girls, may partly contribute to the high levels of overweight/obesity reported in South African adults, particularly in women [27]. The level of central obesity in adolescent girls indicates risk of later metabolic disease. This is of major public health importance as South Africa is undergoing an epidemiologic transition with chronic non-communicable diseases associated with obesity contributing markedly to the burden of disease in this community and other parts of South Africa- despite the burden due to infectious diseases and undernutrition [53-55]. At national level, undernutrition may ultimately affect the gross domestic product through lowered economic productivity - malnourished children are more likely to have poor educational outcomes leading over time to lower incomes, higher fertility, and suboptimal care for their children, thereby contributing to the intergenerational transfer of poverty [2,24].

In conclusion, child growth and nutrition in rural South Africa is clearly shifting along the rural-urban continuum and is tending towards an urban-like profile. Persisting prevalence of undernutrition, particularly stunting, at an early age suggests inadequate interventions to address food insecurity and undernutrition. It also indicates possible need to intervene during the perinatal period. The prevalence of substantial levels of overweight and obesity in the same community presents a multifaceted policy and programme challenge. It is likely that this profile relates to changes in nutrition and dietary patterns, but variation in other factors such as infectious disease burden and physical activity including exercise, as well as social influences, need to be investigated. We therefore recommend a scaling up of research and programme evaluation in order to inform policy on effective intervention strategies that can address the double-pronged problem of malnutrition.

## Abbreviations

HDSS: Health and socio-demographic surveillance system; AIDS: Acquired immune deficiency syndrome; BMI: Body mass index; CDC: Centers for Disease Control and Prevention; Cm: Centimetre(s); DALYs: Disability-adjusted life years; DoH: Department of Health; HAZ: Height-for-age z-scores; HIV: Human immunodeficiency virus; Kg: Kilogram(s); IOTF: International Obesity Taskforce; LMICs: Low- to middle-income countries; M: Metre(s); MDGs: Millennium development goals; NFCS: National food consumption survey; NCHS: National Center for Health Statistics; SAVACG: The South African Vitamin A Consultative Group; UK: United Kingdom; USA: United States of America; USD: United States dollar; WAZ: Weight-for-age z-scores; WHO: World Health Organization; WHtR: Weight-for-height ratio; WHZ: Weight-for-height z-scores; YRBS: Youth risk behaviour survey.

## Competing interests

The authors declare that they have no competing interests.

## Authors' contributions

EWK-M: Design of the study, project management, training and supervising fieldworkers, data management and analysis, writing of the manuscript. Read and approved the final manuscript. KK: Design of the study, overall project co-ordination, reviewing of the manuscript. Read and approved the final manuscript. JMP: Design of the study, review of the manuscript. Read and approved the final manuscript. SMT: Design of the study, review of the manuscript. Read and approved the final manuscript. DD: Design of the study, review of the manuscript. Read and approved the final manuscript. FXG: Design of the study, training of field workers, implementation and supervision of field work, review of the manuscript. Read and approved the final manuscript. SAN: Design of the study, overall project management, reviewing of the manuscript. Read and approved the final manuscript.

## Pre-publication history

The pre-publication history for this paper can be accessed here:

http://www.biomedcentral.com/1471-2458/10/158/prepub
